# Correspondence

**Published:** 1879-10

**Authors:** 


### Correspondence.

Baden-Baden, Aug. 6, 1879.

The American Dental Society of Europe, closed its seventh annual session to-day. This was one of the most profitable, as well as most pleasant of its meetings.

The object of this Society is two-fold: Primarily, progress; this is an essentially progressive profession; Secondarily—recreation from practice.

American Dentists as a whole are generally acknowledged to be in advance of the dentists of other nations; this place of the front is chiefly due to the influence of Dental Societies, there being one or more in each state, in which the best—the foremost men in the profession meet to discuss the different points of theory and practice, giving and receiving new ideas, and by these frequent meetings, stimulating and encouraging each other in this most arduous but interesting profession. American Dentists on the Continent have a high reputation; the name alone has become so popular, that native dentists make use of it, to their own great advantage. Prior to 1873, no Society of American Dentists existed on the Continent. In that year, on the 4th of July, four gentlemen met on the Rigi, and organized a Society, which now numbers more than fifty members from all parts of Europe.

At this seventh meeting, were present the following members: Drs. Abbot, Page and Sylvester, Berlin; Drs. Foster, & Miller, Berlin; Dr. Patton, Cologne; Dr. Cohn, Hamburg; Dr. Sach, Breslau; Dr. Crane, Paris; Dr. Kingsley, Paris; Dr. Barrett, Paris; Dr. Pritchard, Greenleaf, London; Dr. Perkins, London; Dr. Parsons, Bristol; Dr. Jenkins, Dresden; Dr. Van Marter, Florence; Dr. Doremus, Zurich; Dr. Wright, Basel; Drs Williams & Blount, Geneva.

Subjects of interest to the profession were freely and thoroughly discussed.

The Officers for the coming year were elected:

N. W. Williams, President.

Chas. Kingsley, Vice-President.

J. W. Crane, Recording and Corresponding Secretary.

C. M. Wright, Treasurer.

The Society adjourned to meet at Lucern, in August, 1880.

A very ingenious instrument—not yet perfected—was shown to the Society by Dr. Jenkins of Dresden. It is the invention of a German electrician.

The object of this invention is to make use of the electric light in filling teeth; by it the mouth will be so illuminated, that every part will be distinctly visible, and the teeth rendered almost transparent. X.

---

*Editor of Dental Register:—Dear Sir:*—I wish to ask through your valuable Journal, as to the utility of aluminum for dental plates. As a metal, it is less than one-fifth the weight of gold; it is a good conductor of heat, and is very ductile. Is it durable in the mouth? and if not, in what way does it fail? If it has proved valuable for this purpose? what is the best method of using it? Is casting it practicable in the hands of the average dentists? or in the hands of any one? If so, how is it done? Is it easily swedged, and is not this the better method?

What is the best method of attaching the teeth to such a plate? STUDENT.

